# A chloroplast structured photocatalyst enabled by microwave synthesis

**DOI:** 10.1038/s41467-019-09509-y

**Published:** 2019-04-05

**Authors:** Shuning Xiao, Dieqing Zhang, Donglai Pan, Wei Zhu, Peijue Liu, Yong Cai, Guisheng Li, Hexing Li

**Affiliations:** Key Laboratory of Resource Chemistry of Ministry of Education, Shanghai Key Laboratory of Rare Earth Functional Materials, College of Chemistry and Materials Science, Shanghai Normal University, 200234 Shanghai, China

## Abstract

Photosynthesis occurs through the synergistic effects of the non-ncontinuously distributed components in the chloroplast. Inspired by nature, we mimic chloroplast and develop a generic approach to synthesize non-continuously distributed semiconductors threaded by carbon nanotubes. In the synthesis, carbon nanotubes serve as microwave antennas to produce local super-hot dots on the surface, which might induce and accelerate various organic/inorganic semiconductors assembly. With the unique nanoscale designed bionic architecture, a chloroplast structured photocatalyst with 3−dimentional dual electron transfer pathways facilitate enhanced photocatalytic performance. The as-synthesized carbon nanotubes-titanium oxide achieves a record-breaking efficiency of 86% for nitric oxide treatment under ultraviolet light irradiation. As a general strategy, a wide variety of carbon nanotubes threaded chloroplast structured nanomaterials can be synthesized and these nanomaterials could find applications in energy chemistry, environmental science and human health.

## Abstract (web summary)

Inspired by the favorable configuration of chloroplast, here the authors develop a microwave aided approach for the synthesis of similarly structured photocatalysts. The non-continuously distributed TiO_2_ nanomaterials threaded by carbon nanotubes enable a high efficiency for nitric oxide treatment.

## Introduction

**Fig. 1:**
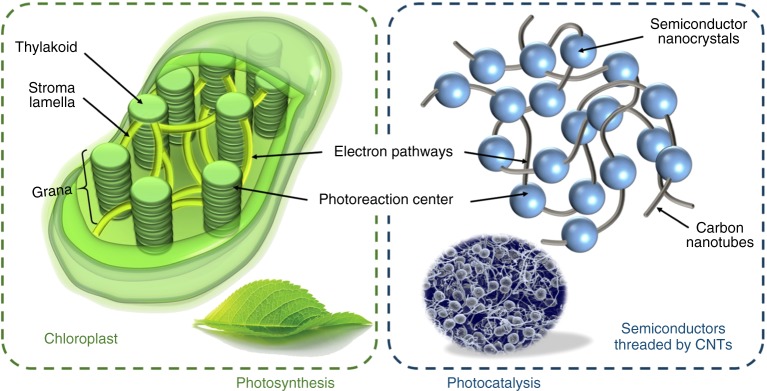
The design principle of the chloroplast structured catalyst. Top view of chloroplast structure (left) and the architecture of the non-continuously distributed semiconductors threaded by CNTs (right)

Nano-sized semiconductors have been widely used as electrodes and photocatalysts in the application of energy and environmental field^1–4^. However, without a favorable structure, they tend to suffer from some disadvantages such as the ease of aggregation and rapid photoelectron-hole recombination^5–8^. The photosynthesis allows plants to harvest solar energy by converting it into carbohydrate molecules to fuel all biological life activities on Earth^9–11^. Typically, photosynthesis efficiently occurs in the chloroplast (Fig. 1 left) with the nature designed non-continuously distributed components: the *Thylakoid* assembled by disk-like *Grana* is the site where light-dependent reactions take place and the layer-like *Stroma Lamella* links each *Thylakoid* by providing electron transport channels. Inspired by nature, great efforts have been devoted to designing binary, ternary, and multi-component hierarchical nanocomposites with synergistic effects^12–17^. Among them, the carbon nanotubes (CNTs) are widely used since they usually display fascinating electrical and optical properties endowed by the unique 1-dimensional properties. Such properties are expected and advantageous for the surface reactions and charge transport processes^18–22^.To decorate CNTs with semiconductors, the in-situ solvothermal strategy is reported^21,23–25^. However, it is still hard to selectively control the growth sites of semiconductors on the CNTs and it is also difficult to avoid the free nucleation and growth. As we know, people can hardly realize the intimate contact between CNTs and nanocrystals.

We consider introducing microwave heating into the in situ fabrication involving CNTs, aiming to obtain the chloroplast structured photocatalysts enabled by microwave synthesis (Fig. 1 right). We infer that CNTs can selectively absorb microwaves and preferentially convert microwave energy into thermal energy with the formation of local super-hot dots, thus allowing and accelerating various organic/inorganic chemical reactions that occur on the CNTs surfaces. Because the geometry of CNTs presents a notably high aspect ratio of greater than 1000, the contacts between the ends of the long tubes are sufficient to create a conducting network. The remarkable electrical conductivity of CNTs at microwave frequencies also facilitates their use as broadband microwave-absorbing materials^26^. As a typical example, the concept of solar-energy convertor by constructing nano-rectenna solar cells using aligned CNTs as optical antennas is also introduced^27^.

Here, our design is that CNTs would work as microwave antennas in the liquid–solid interficial synthesis. By using CNTs as microwave antennas, we have developed a general strategy for in-situ fabrication of semiconductors non-continuously threaded by the CNTs. As show in Fig. 1 (right), the synthesis was based on the idea of deploying 1-dimensional CNTs as a direct-charge transport superhighway. Taking advantage of the direct growth of photo-active semiconductors onto CNTs, a 3−dimentional conductive network with dual electron transfer pathways was obtained, which was very similar to the photosynthesis process in chloroplast. We have chosen one chloroplast structured photocatalyst of the non-continuously distributed and well-assembled single-crystal titanium oxides threaded by CNTs (CNT-TiO_2_) to study their microwave synthesis mechanism, as well as their photocatalytic performances in nitric oxide oxidation reaction. This unique structure is proved for promoted light harvesting ability, while the 3−dimensional CNTs networks provided electron transfer pathways for accelerating chemical reactions. Thus, the as-synthesized CNTs-TiO_2_ present a record-breaking efficiency of 86% for ultraviolet light-driven nitric oxide abatement. In addition, as a general strategy, a series of chloroplast structured nanomaterials can be synthesized and should be potentially used in the energy and environment fields.

## Results

### Morphology of the chloroplast structured photocatalyst

**Fig. 2:**
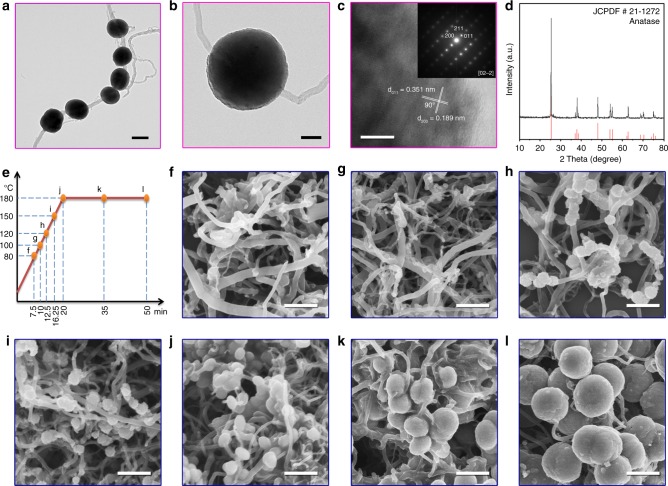
Morphology and phase structure of chloroplast structured CNT-TiO_2_. TEM images (**a**, **b**), HRTEM image with attached SAED pattern (**c**), and XRD pattern (**d**) of the as-prepared CNT-TiO_2_. Different CNT-TiO_2_ samples prepared at desired microwave-heating time and temperature (**e**) and their corresponding FESEM images (**f**–**l**). Scale bars in (**a**): 500 nm, (**b**): 100 nm, (**c**): 2 nm, and (**f**–**l**): 500 nm

As a typical example, the CNT-TiO_2_ was synthesized by heating the dimethyl sulfoxide (DMSO) solution containing TiCl_3_ and CNTs with microwave. Transmission electron microscopy (TEM) images (Fig. 2a, b) demonstrated that the as-received sample consisted of uniform microspheres with an average diameter of approximately 500 nm threaded by CNTs. Both high-resolution transmission electron microscopy (HRTEM) and selected-area electron diffraction (SAED) images (Fig. 2c and inset) confirmed the single-crystalline nature of TiO_2_ on the basis of well-resolved diffraction dots and the corresponding lattice spacing. Furthermore, the X-ray diffraction pattern (Fig. 2d) displayed sharp diffraction peaks characteristic of the pure anatase TiO_2_ phase (JCPDF card No. 21–1272).

To examine the formation mechanism of such chloroplast structure, the CNT-TiO_2_ was prepared by time-temperature-programmed process (Fig. 2e) and different samples obtained at desired heating times and temperatures were collected. The field emission scanning electron microscope (FESEM) images were shown in Fig. 2f–l. At the initial stage (microwave heating 7.5 min to 80 °C), only CNTs were observed in the FESEM images (Fig. 2f), indicating that TiO_2_ could not be formed on CNTs below 80 °C. As shown in Fig. 2g, when the microwave heating time was extended to 10 min with temperature reaching upto 100 °C, small nanoparticles were deposited onto the CNTs surface due to the formation of TiO_2_ nanocrystals. Figure 2h–j revealed that further increasing microwave heating time to 20 min with the temperature reaching upto 180 °C resulted in more TiO_2_ nanocrystals onto the CNTs surface which is determined as the crystal nucleation process. By keeping at 180 °C, increasing the microwave-heating time from 0 to 30 min caused significant increase in the particle size of TiO_2_ nanocrystals from Fig. 2j–l.

It is worth mentioning that this synthesis method is generic and can be applied to different materials. A series of nanocomposites with chloroplast structure has been prepared including CNT-CeO_2_, CNT-Mn_3_O_4_, CNT-Cu_2_O, CNT-ZIF-67, and CNT-ZIF-8 (see the FESEM images in Supplementary Fig. 1 and the XRD patterns in Supplementary Fig. 2).

### Photocatalytic removal of nitric oxide

**Fig. 3:**
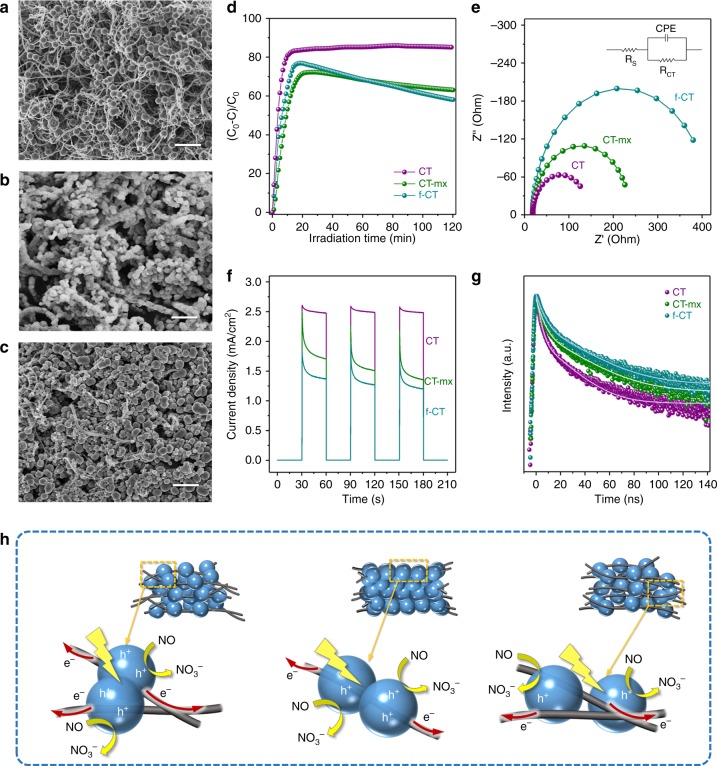
Chloroplast structure of CNT-TiO_2_ catalysts facilitated the electron transport and enhanced the photocatalytic NO oxidation performance. SEM image of different CNT-TiO_2_ composites: (**a**) CT, (**b**) f-CT and (**c**) CT-mx. **d** UV-light-driven photocatalytic NO oxidation performances, (**e**) electrochemical impedance spectra (EIS) with the equivalent circuit model in the inset, (**f**) photocurrent spectra and (**g**) time-resolved transient photoluminescence decay profiles of sample CT, f-CT, and CT-mx. **h** The scheme illustration of the morphologies and photo-carries separation process of sample CT, f-CT, and CT-mx. Scale bars in **a**–**c**: 2 μm

As CNTs could promote electron transport in nanocomposites, they could work as co-catalysts or additives and should have considerable applications in a series of fields^28–32^.The CNT-TiO_2_ microspheres (CT, shown in Fig. 3a) were used as catalysts in the photocatalytic NO oxidation to evaluate the advantages of this unique chloroplast structure. Besides, the completely encapsulated core-shell structure of CNT-TiO_2_ (f-CT, shown in Fig. 3b), as well as the mechanical mixing of CNTs and TiO_2_ microsphere (CT-mx, shown in Fig. 3c) could also be prepared as the references. As illustrated in Fig. 3d, in the gas-phase flow photocatalytic NO oxidation reaction, the CT sample showed a remarkable high NO conversion rate of ~86% under UV-light irradiation and desirable reaction stability. To the best of our knowledge, this is the record-breaking photocatalytic NO conversion among all the UV-response catalysts (e.g., O_v_-TiO_2_ at ~20%^33^, single atom Pd-TiO_2_ at ~47%^34^, g-C_3_N_4_-TiO_2_ at ~56%^35^, PtO_x_-TiO_2_ at 71%^36^ and C, N-doped P25 TiO_2_ at 79.6%^37^) While, the f-CT exhibited the lower conversion of ~77% after equilibrium in 20 min, but gradually dropped to ~58% after 120 min irradiation, which was even much lower than that for sample CT-mx (~72%). Considering that these samples own the similar CNT/TiO_2_ ratio, surface area and light absorption properties analyzed in Supplementary Fig. 3, the difference in photocatalytic performance could be considered as their different charge transport properties. Thus, the photocurrent, electrochemical impedance tests, charge dynamic analysis, as well as the scheme illustration were shown and discussed in Fig. 3e–h to understand the photo-excited carriers’ separation and transport processes. For the CT and f-CT, microwave synthesis created an intimate contact between CNTs and TiO_2_ by the covalent bonds of Ti–O–C=O or Ti–O–C (located at ~1120 cm^−1^) formed due to the etherification process between the CNTs surface carboxyl/hydroxyl groups and TiO_2_, which was evidenced by the Fourier transform infrared (FT-IR) spectra as shown in Supplementary Fig. 4. With this kind of strong “line-contact” (shown in Fig. 3h, left and middle) rather than the weak “point-contact” in CT-mx (shown in Fig. 3h right), the photo-excited electron could be separated rapidly, thus resulting in the remarkably high performances in NO removal and high densities of photocurrent. The electrochemical impedance data (EIS) were simulated base on the Randles equivalent circuit model (shown in Fig. 3e and the inset). The circuit included a solution resistance (R_s_), charge transfer impedance (R_CT_) and a non-ideal capacitor (CPE), which substituted the double layer capacitance of the electrode–electrolyte interface. The capacitor is related to the adsorption of reactant on the surface of electrode and the diffusion process associated with charge transfer reaction across the electrode–electrolyte interface. The charge transfer impedance (R_CT_) of CT, CT-mx, and f-CT were distinguished and could be fitted to 126.4, 218.9, and 400.2Ω, respectively. For the CT and CT-mx samples, they exhibited lower R_CT_ value in the EIS spectra and relatively higher photocurrents shown in Fig. 3f. This is attributed to 3-dimensional conductive networks in topologies constructed by the crossing linked CNTs (shown in Fig. 3h left) which promoted a better electric conductivity and provided index increasing pathways for independent electron transport. Meanwhile, the time-resolved transient photoluminescence decay profiles were shown in Fig. 3g (detail fitted parameters shown in Supplementary Table 1). The average emission life-time of CT (9.2 ns) was shorter than that of CT-mx (13.6 ns) and f-CT (14.8 ns). The observed difference in mean lifetimes was due to the number of effective interfaces between TiO_2_ and CNTs. For the sample of CT, multiple interfaces between TiO_2_ and CNTs provided relatively efficient electron transfer resulting in a short lifetime. While, the aggregation of TiO_2_ particles might suppress the interfacial electron transfer from TiO_2_ to CNT, thus resulting in the prolonged lifetime for the f-CT sample. Furthermore, the photocarrier density calculated from the Mott–Schottky plots (Supplementary Fig. 5) also confirmed that such chloroplast structure successfully prolonged the life-time of photogenerated carriers, thus presenting enhanced activities in NO oxidation. However, in sample f-CT, there existed less interaction between each CNTs as a result of conductivity decrease because the CNTs were completely wrapped by semiconductor TiO_2_ (as shown in Fig. 3h middle). Although the sample f-CT showed the high activity and high photocurrent in the initial stage, the lack of topological conductive networks finally resulted in the rapid recombination of photo-carriers further led to their dramatically drop in the long run. Therefore, we think different photogenerated charge kinetics over various samples also caused the different selectivity of NO_3_^−^ and NO_2_ product in the NO oxidation reaction. Such hypothesis was supported by Supplementary Fig. 6, which showed the concentration profiles of NO and NO_2_ during the photocatalytic NO oxidation reaction when different catalysts were applied. After analyzing the real-time NO and NO_2_ concentration, the NO_2_ selectivity in 2 h could be 4.5%, 6.8%, and 1.2% for CT-mx, f-CT, and CT, respectively, indicating that fast carrier separation kinetics over the CT sample promoted the high concentration of reactive holes to realize deeply oxidation of NO to NO_3_^−^ instead of NO_2_.

### The formation of localized “super-hot” dots on CNTs

**Fig. 4:**
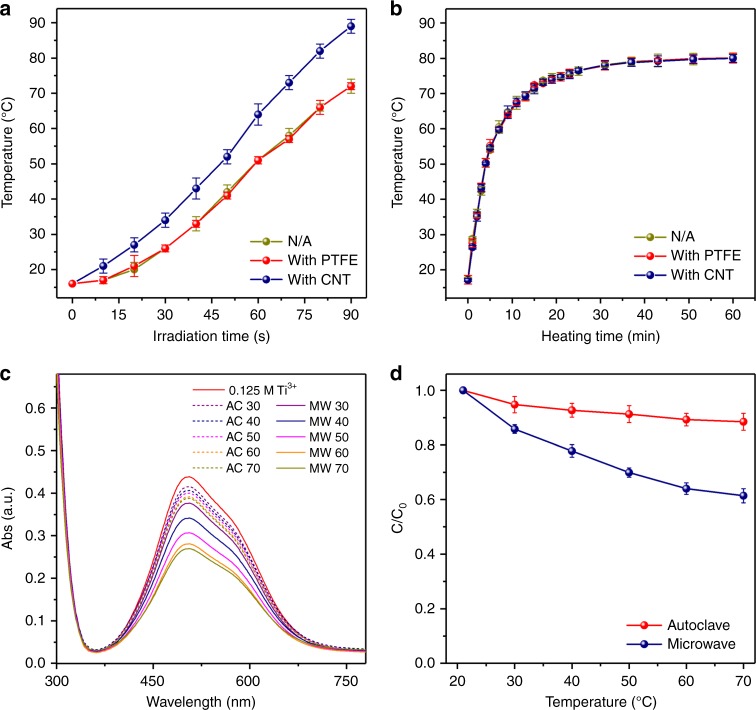
Microwave effects on CNTs. Plots of the temperature of DMSO solution (200 mL) vs. heating time in the presence or absence of CNTs and PTFE (100 mg) with the heating source of (**a**) microwave irradiation (800 W) and (**b**) oil bath (90 °C). The Ti^3+^ adsorption properties of CNTs: (**c**) UV-Vis spectra of TiCl_3_ solution and (**d**) temperature-dependent concentration change of Ti^3+^ under microwave irradiation and autoclave hydrothermal heating by keeping the specific temperature for 20 min

The CNTs played a key role in fabricating chloroplast structured nanomaterials. As well-known, the loss tangent (tan *δ*) is an intrinsic parameter used to define the ability of a specific material or solvent to convert microwave energy into heat^38^. The tan *δ* value can be expressed as tan *δ* = ε”/ε’, where *ε*’ refers to the polarizability and *ε*” is the dielectric loss corresponding to the efficiency to convert the microwave energy into heat^39,40^. Different materials exhibit different tan *δ* at a given microwave frequency and temperature. The CNTs have an excellent high tan *δ* of 1.45 at a frequency of 2.45 GHz and room temperature^41^, which is much more positive than that of many solvents (e.g., acetone = 0.054, water = 0.123, DMF = 0.161, methanol = 0.659, and DMSO = 0.825)^38^. Thus, the CNTs could act as microwave antennas to efficiently absorb microwave and subsequently convert microwave energy into thermal energy, leading to the formation of surface “super-hot” dots with much higher temperature than that of bulk solution^42–45^. Unfortunately, we could not directly measure the local surface temperature to confirm the “super-hot” dots on CNTs. Under this case, we determined the DMSO solution temperature by heating the system in the presence or absence of CNTs and observed the variation of the solution temperature. As shown in Fig. 4a, the DMSO solution containing CNTs exhibited a substantially fast heating rate that resulted in a higher solution temperature than that of the pure DMSO solution. However, the solution containing polytetrafluoroethylene (PTFE) with low tan *δ* (~0.002 at 2.45 GHz) exhibited nearly the same temperature as the pure DMSO solution. The enhanced heating rate of solution in the presence of CNTs was caused by the heat transfer from CNTs to solution based on the much higher temperature on the CNTs surfaces. In contrast, when the regular oil-bath heating was used instead of microwave, the DMSO solution containing CNTs or PTFE exhibited the similar temperature as the pure solution (see Fig. 4b). It indicated that traditional heating transfer could hardly be affected by the additives in solution. To sum up the above, the CNTs with much higher tan *δ* could work as microwave collectors and converters in solution via the localized “super-hot” dots under the specific electromagnetic wave irradiation which facilitated the localized nucleation and growth of TiO_2_ crystal on CNTs instead of solution during the synthesis.

### Designated adsorption of metal-ions on CNTs

To form the chloroplast structures, the localized Ti^3+^ adsorption was also important. As is confirmed by the metal-ion adsorption test in Fig. 4c, d, we also believed the microwave favored the adsorption of Ti^3+^ by oxygen-contained functional groups (carboxyl or hydroxyl) on CNTs from the bulk solution. UV-Vis was used to track the concentration of Ti^3+^ since the absorbance in the 505 nm wavelength is the standard Ti^3+^ signal. Figure 4c demonstrated the UV-Vis spectra of 0.125 M TiCl_3_ solution before and after keeping on the temperature from 30 °C to 70 °C for 20 min under both microwave irradiation and regular hydrothermal autoclave. The corresponding temperature-dependent solution concentration change was shown in Fig. 4d, which clearly exhibited that the CNTs showed higher Ti^3+^ adsorption capability under microwave heating than that under regular hydrothermal heating at the same temperature. Meanwhile, increasing microwave heating temperature (more microwave energy absorbed by CNTs) leaded to the significant enhance in Ti^3+^ adsorption since the concentration of Ti^3+^ solution dropped from 85.5% (30 °C) to 61.4% (70 °C). However, in the autoclave hydrothermal condition, the adsorption amount just showed a slightly enhance with increasing the temperature. Thus, microwave could also enhance the ability for CNTs to adsorb metal-ions and induce their localized hydrolysis on the surface of CNTs. In addition, the CNTs also lowered the nucleation temperature of TiO_2_ crystal. As shown in Supplementary Fig. 7, by microwave heating TiCl_3_ in DMSO solution for 10 min to 100 °C, the solution remained original yellow color and no significant solid products were found. It suggested that the hydrolysis reaction of TiCl_3_ could not occur at such low temperature in this case. On the contrary, in the similar process with CNTs added, the yellow solution became much lighter, accompanied by the formation of gray powders at the bottom. The gray powder can be further confirmed by the FESEM image of Supplementary Fig. 8a. On the surface of the CNT, the bright spots in red circles may be the nuclei of TiO_2_ crystal. Meanwhile, we also found that, when the TiCl_3_ hydrolysis reaction was carried out at 100 °C via regular oil-bath heating, no significant reaction occurred even in the presence of CNTs as shown in the Supplementary Fig. 7 and Supplementary Fig. 8b because no surface change could be observed. These results demonstrated that, under microwave heating, the temperature of the CNTs’ surface should be much higher than that in the bulk solution and was sufficiently high for the hydrolysis of Ti^3+^ into TiO_2_. Thus, the TiO_2_ nanocrystals formed only on the CNTs surface rather than in the bulk solution. To further confirm this hypothesis, we also used a 1-dimensional polytetrafluoroethylene (PTFE) with a very low tan *δ* as a reference material instead of CNTs. From the FESEM of PTFE-TiO_2_ (shown in Supplementary Fig. 9), we observed that only very few TiO_2_ nanocrystals growing on the PTFE surface after the precursor was microwave-heated to 180 °C and maintained for 30 min. This was obviously due to PTFE’s poor ability for absorbing microwave energy to generate local “super-hot” dots, while the CNTs surface could initiate the hydrolysis of adsorbed Ti^3+^ due to the localized high temperature, leading to the in situ formation and assembly of TiO_2_ nanocrystals along them.

### The role of CNTs surface functional group

**Fig. 5:**
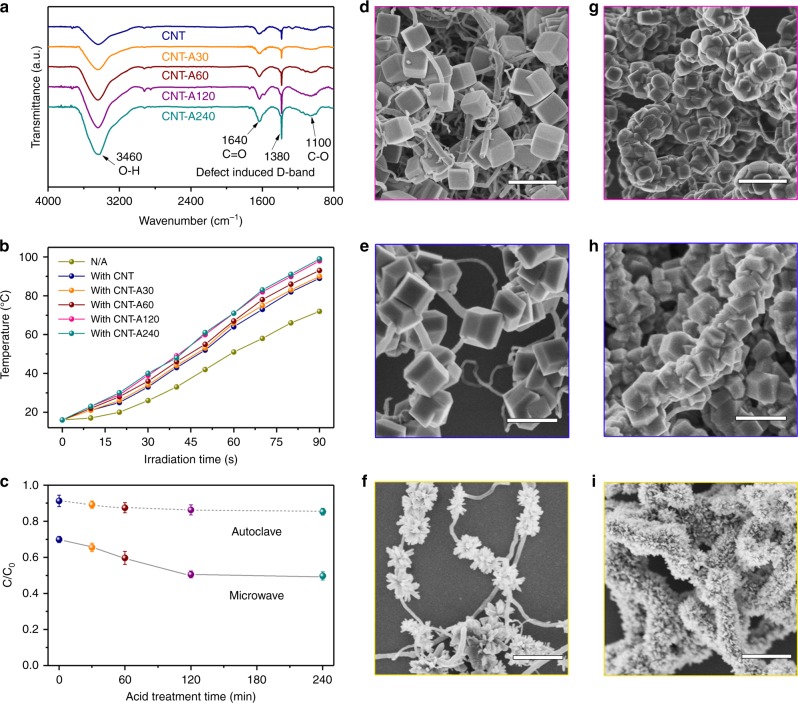
Surface functional groups affected the microwave antenna performance of CNTs. **a** FT-IR spectra of CNTs after HNO_3_ pre-treatment for 0, 30, 60, 120 and 240 min. **b** Plots of the temperature of DMSO solution (200 mL) vs. microwave irradiation time in the presence or absence of different CNTs. **c** CNTs acid pre-treatment time-dependent concentration change of Ti^3+^ under microwave irradiation and autoclave hydrothermal heating by keeping the heating temperature of 50 °C for 20 min. **d**–**i** FESEM images of (**d**, **g**) CNT-anatase TiO_2_nanocubes with exposed (001) and (010) facets, (**e**, **h**) CNT-threaded anatase TiO_2_nanodecahedrons with exposed (001) and (101) facets, and (**f**, **i**) CNT-threaded rutile TiO_2_ nanoflowers by using (**d**–**f**) untreated and (**g**–**i**) CNTs pre-treated with HNO_3_ for 240 min. Scale bars in **d**–**i**: 1 μm

The presence of polar groups like carboxyl (–COOH) and hydroxyl (–OH) on CNTs also played a vital role in this microwave reaction mainly from the following two aspects: first, oxygen-contained groups promoted the microwave absorbance and conversion of CNTs due to the enhanced dielectric property by increased polarizability which lead to the formation of more “super-hot” dots in the Joule heating process;^46^ second, the surface –COOH and –OH could work as the active absorption sites for metal-ions to ensure the localized nucleation and growth of nanocrystals. According to the phenomenological model^47^, the CNTs might undergo a highly efficient superheating process through transformation of electromagnetic energy into mechanical vibrations because of the presence of polar groups connected with the six-membered-ring carbon backbone to form dangling bonds. These dangling bonds, together with the CNTs, resulted in the enhanced absorbance of microwaves and the subsequent conversion of the absorbed microwave energy into thermal energy, leading to the abrupt increase of the CNTs’ surface temperature. It could be confirmed by comparing the solution heating rate by adding CNTs with different acid treatment time (detailed method described in the supporting information). As the Fourier-transform infrared (FT-IR) spectra (Fig. 5a) revealed, the amounts of –COOH and –OH groups covalently bonded to the CNTs increased with the prolonging of HNO_3_-pretreating time from 30 min to 240 min. As shown in Fig. 5b, more oxygen-contained group resulted in the significantly increasing heating rate of the solution. Moreover, pre-treatment enhanced the metal-ion adsorption illustrated in Fig. 5c. By fixed the microwave/autoclave temperature in 50 °C, the Ti^3+^ concentrations remaining in solution were compared. Although the acid treatment on CNTs enhanced the Ti^3+^ adsorption by regular autoclave hydrothermal to a certain degree, the increasing rate by microwave was much more considerable and obvious. Interestingly, the CNT-A120 and CNT-A240 presented the similar temperature raising phenomenon, as well as ion adsorption property. It was because that the surface functional group tended to be saturation after 120 min of pre-treatment, thus further acidification had less effect on the surface property of CNTs.

Accordingly, by well controlling the surface oxygen-contained groups, we could continuously adjust the density of TiO_2_ nanospheres threaded by CNTs from the non-continuous distribution state to the completely encapsulated core-shell structure shown in Supplementary Fig. 10. In addition, other CNT-TiO_2_ nanocomposites with hierarchical morphologies could also be synthesized based on the present microwave-antenna strategy (see the experimental details in the Supporting Information), showing its universality. The FESEM images in Fig. 5d–i displayed anatase TiO_2_ nanocubes with exposed (001) and (010) facets, anatase TiO_2_ nanodecahedrons with exposed (001) and (101) facets, rutile TiO_2_ nanoflowers (see the XRD patterns in Supplementary Fig. 11) threaded by untreated CNTs (d) (e) (f) and CNTs with acid treated for 240 min (g) (h) (i). We also compared the FESEM images of the TiO_2_ in anatase microspheres, rutile nanoflowers and anatase nanodecahedrons obtained via autoclave hydrothermal and microwave route in the presence or absence of CNTs, respectively (see Supplementary Fig. 12). When regular autoclave hydrothermal heating was used, the TiO_2_ obtained with and without CNTs exhibited similar morphologies, and no direct growth of TiO_2_ nanoparticles on the CNTs’ surface was observed. This was due to the autoclave conduction heating mode which made the solution temperature to be higher than that of CNTs in the initial stage since the heat was transferred from the outside to the inside in the autoclave system. Therefore, the nucleation site could be in the solution rather than on the CNTs’ surfaces. However, with the application of microwave instead of regular hydrothermal heating, the TiO_2_ samples obtained with and without CNTs exhibited remarkably different morphologies. In the absence of CNTs, only randomly dispersed TiO_2_ nanoparticles were observed in the solution. Only by satisfying the two conditions of microwave heating and the presence of CNTs, the chloroplast structure nanohybrids could be prepared. These results further confirmed the nature of CNTs working as microwave antenna, converting more energy into the heat and being the nucleation sites for direct growth of TiO_2_ crystals. At the same time, because of the CNTs’ outstanding electrical and mechanical characteristics, such as their high surface area, desirable volume ratio and the intrinsic metallic character (CNT larger than 2 nm in diameter), they could also efficiently avoid aggregation of the TiO_2_ nanoparticles, corresponding to a uniform shape with small particle size compared with those obtained in the absence of CNTs^48^.

## Discussion

**Fig. 6:**
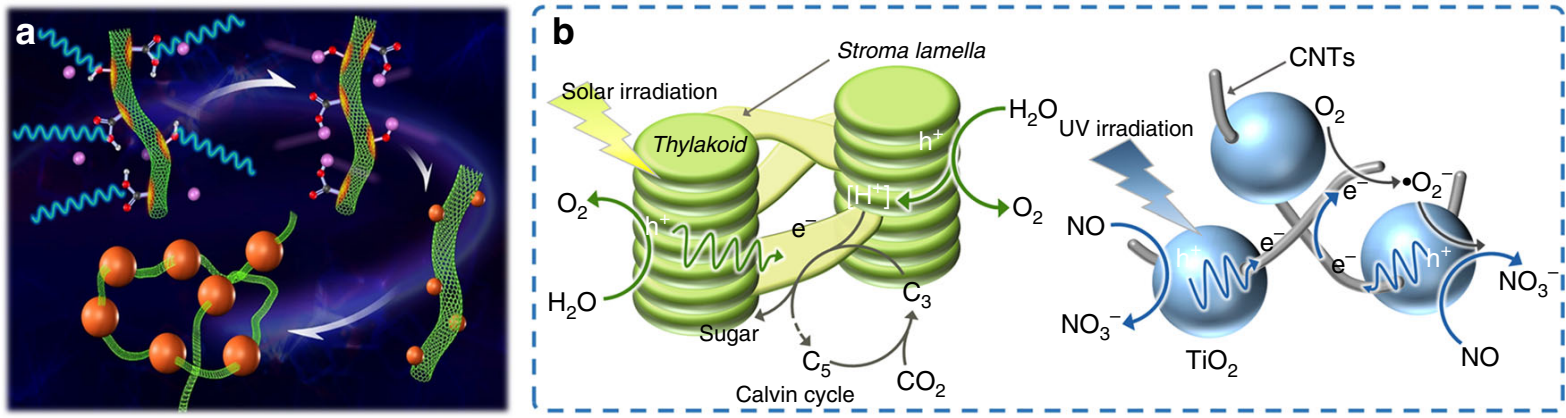
The mechanism illustration for CNTs as microwave antennas and chloroplast structures in photosynthesis as well as photocatalysis. **a** Schematic illustration of the CNT-TiO_2_ preparation process: microwave absorbing and converting into super-hot dots (red dots), Ti^3+^ ions (purple dots) adsorption onto super-hot dots, Ti^3+^ hydrolysis into TiO_2_ crystal seeds (small brown dots), and TiO_2_ nanocrystals (big brown dots). **b** The photosynthesis mechanism of chloroplast (left) and the photocatalytic NO removal mechanism of chloroplast structured CNTs-TiO_2_

At this point, we could summarize the microwave antennas mechanism of the in situ fabricating chloroplast structured CNTs-threaded TiO_2_ nanocrystals illustrated in Fig. 6a. At the initial stage, the CNTs could strongly absorb electromagnetic wave like microwave antennas and then convert the microwave energy into heat to generate “super-hot” dots on their surface (red dots). These “super-hot” dots favored adsorption of Ti^3+^ ions (purple dots) by carboxyl or hydroxyl groups. When the surface “super-hot” dots reached the nucleation temperature, the Ti^3+^ could hydrolyze into nuclei seeds (small brown dots) on the CNTs. Finally, with the increase of microwave time and temperature, more “super-hot” dots on the CNTs surface generated, which could absorb more Ti^3+^ ions, followed by hydrolyzing into more nuclei seeds of TiO_2_. The nuclei further grew into TiO_2_ nanocrystals (big brown dots) and finally formed the chloroplast structure. In this synthesis of CNTs-TiO_2_ nanocomposites, after microwave-heating for 20 min with the corresponding temperature in 180 °C, the density of TiO_2_ nanocrystals on the CNTs surface reached maximum. Further increasing microwave-heating time, the temperature could not increase the adsorption of Ti^3+^ ions onto CNTs surface due to the spatial hindrance. Further prolonging the microwave-heating time resulted in crystal growth, leading to the increased size of TiO_2_ nanocrystals as shown in Fig. 2j–l.

By mimicking the photosynthesis process in chloroplast, the as-obtained chloroplast structured CT sample exhibited the highest and most stable photocatalytic NO conversion performance, and its photocatalytic mechanism was similar with the photosynthesis in chloroplast. As illustrated in Fig. 6b, in photosynthesis the light-dependent reactions occur in the disk-like *Grana* assembled *Thylakoid*, where H_2_O can be oxidized to O_2_ by the photogenerated holes through photosystem I and II with the formation of proton (H^+^). While, the layer-like *Stroma Lamella* links each *Thylakoid* by providing electron/H^+^ transport channels to promote the Calvin cycle for efficient capturing of CO_2_ to produce sugar. In the photocatalytic NO removal reaction, as the light-dependent reaction, the CT sample with the chloroplast structure can be excited by UV light with generated holes in TiO_2_ microsphere to deeply oxidize NO to NO_3_− (confirmed by the UV-vis analysis in Supplementary Fig. 13). As shown in Supplementary Fig. 13c, the NO_3_− production over the CT sample increased with the irradiation time. As mentioned before, the NO_3_− selectivity could be more than 98% over the sample CT. After 2 h reaction, the accumulated HNO_3_ on the catalyst surface did not affect the stability of the catalyst due to the enough adsorption sites for NO_3_^−^. This also could be ascribed to the fast carrier separation kinetics over the CT sample which could promote the high concentration of reactive holes to release deep oxidation of NO to NO_3_^−^ instead of NO_2_. Furthermore, less NO_2_ adsorption also made this catalyst more stable than the other two catalysts. The intimate “line-contact” between CNTs and TiO_2_ microsphere, as well as the constructed 3-dimensional CNTs conductive networks facilitate the photo-carries’ separation. With the negative reduction potential, the accumulated electrons on the CNTs reacting with O_2_ can induce the formation of •O_2_− species. This was confirmed by the electron spin resonance results as shown in Supplementary Fig. 14. Such active species can further benefit the oxidation reaction to realize the record-breaking performance.

In conclusion, by mimicking chloroplast towards efficient charge transport, we have developed a general microwave-antenna strategy for in situ synthesizing non-continuous distribution of nano-semiconductors threaded by CNTs. The 1-dimensional materials of CNTs with strong ability for absorbing microwave were used as microwave antennas to generate the high-temperature local surface “super-hot” dots. Such “super-hot” dots would induce adsorption, hydrolysis or coordination of metallic ions, followed by nucleation, crystal-growth and self-assembly into a unique architecture with metal oxides and metal organic frameworks (MOFs) nanocrystals threaded by CNTs. In CNT-TiO_2_ nanocomposites, like chloroplast, the cross-linked CNTs provided electron conducting pathways facilitating enhanced photocatalytic performance in the gas-phase reaction. This new microwave-antenna strategy can be extended to in situ fabrication of other 1-dimensional binary or ternary nanocomposites and even 2-dimensional or 3-dimensional chloroplast structured nanocomposites with controllable architectures like those found in nature, which may offer more opportunities for their applications in environmental and energy fields.

## Methods

### Synthesis of CNT-TiO_2_ with anatase microspheres (sample CT)

In a typical synthesis, 20 mg CNTs were dispersed in 18 mL dimethyl sulfoxide (DMSO, Aladdin) for 30 min to form a black suspension in an ultrasonic cleaner. As references, CNTs with different acid treatment time were used instead of the CNT without acid treatment. Then, 2 mL 15 wt% TiCl_3_ aqueous solution with 10–15 wt% HCl (Merck-Schuchardt) was added to the suspension with magnetic stirring for 10 min. Subsequently, the mixture was moved to a 40 ml quartz vessel with a Teflon lid. It was treated at 180 °C for 30 min with a heating rate of 15 °C min^−1^ and with an initial pressure of 35 bar by N_2_ in a single chamber microwave digestion system (Ultrawave, Milestone). The resulting powder was washed with deionized water and absolute ethanol for 3 times, followed by vacuum drying at 80 °C for 4 h.

### Photocatalytic NO oxidation activity test

The photocatalytic NO oxidation in gas phase was carried out at ambient temperature in a continuous flow reactor with volume of 10.8 L (36 × 20 × 15 cm). For UV lights driven photocatalysis, eight of UV lamps (6w, 365 nm) located vertically above the reactor was used as light source. In each run of experiments, an air gas flow containing 500 ppb NO was allowed to pass through 0.20 g photocatalyst at the flow velocity of 4.0 L/min. After reaching adsorption-desorption equilibrium on the photocatalyst, the lamp was turned on to start the photocatalysis reaction. The concentration of NO was continuously measured by using a chemiluminescence NO analyzer (Thermo Environmental Instruments Inc. Model 42i). The NO removal rate (%) was calculated based on the following equation: NO removal rate (%) = (*C*_0_– *C*)/*C*_0_ × 100%, where *C*_0_ and *C* refer to the NO concentration determined before and after reaction.

## Supplementary information

Supplementary Information

## Data Availability

The data that support the findings of this study are available from the corresponding authors upon reasonable request.
