# Supplementary material for: A chloroplast structured photocatalyst enabled by microwave synthesis
Source: Nat Commun. 2019 Apr 5;10:1570. doi: 10.1038/s41467-019-09509-y (PMC6450964; doi:10.1038/s41467-019-09509-y)
Supplement: Supplementary file 1 — Supplementary Information [file 41467_2019_9509_MOESM1_ESM.pdf]

Supplementary Information

A chloroplast structured photocatalyst enabled by microwave synthesis

Xiao et al.

Supplementary Figures

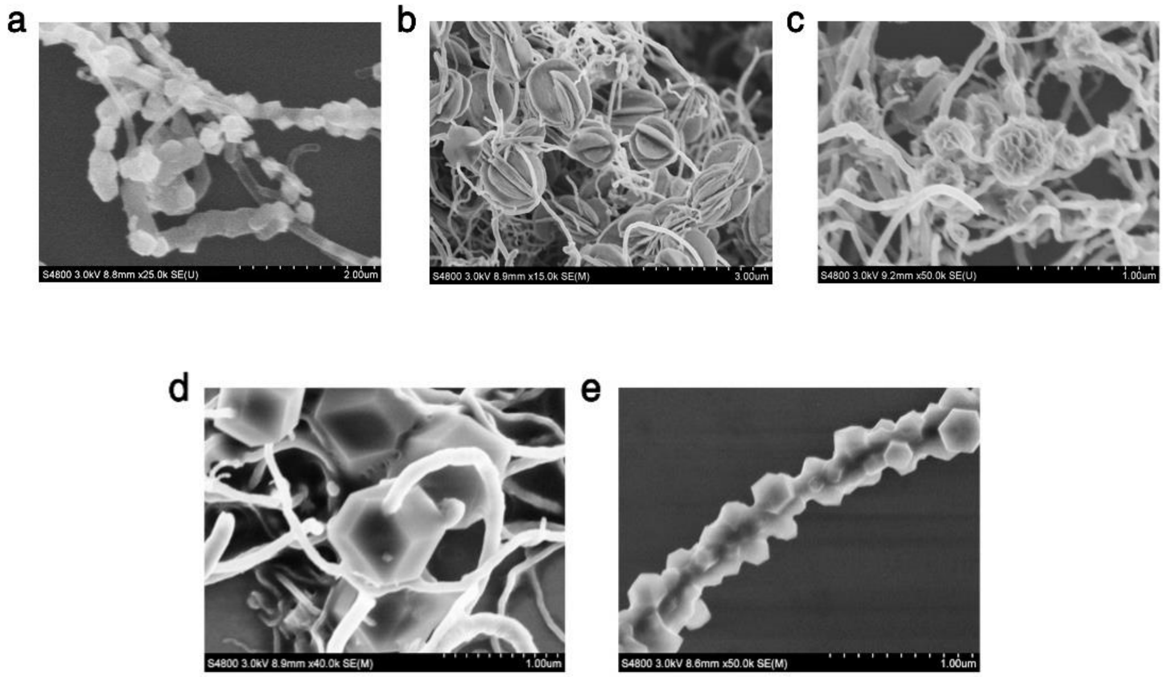

Supplementary Figure 1 The general microwave route to fabricate other CNT/semiconductor composites with unique structure. FESEM images of CNT-CeO_2 (a), CNT-Mn_3O_4 (b), CNT-Cu_2O (c), CNT-ZIF-67 (d), and CNT-ZIF-8 (e).

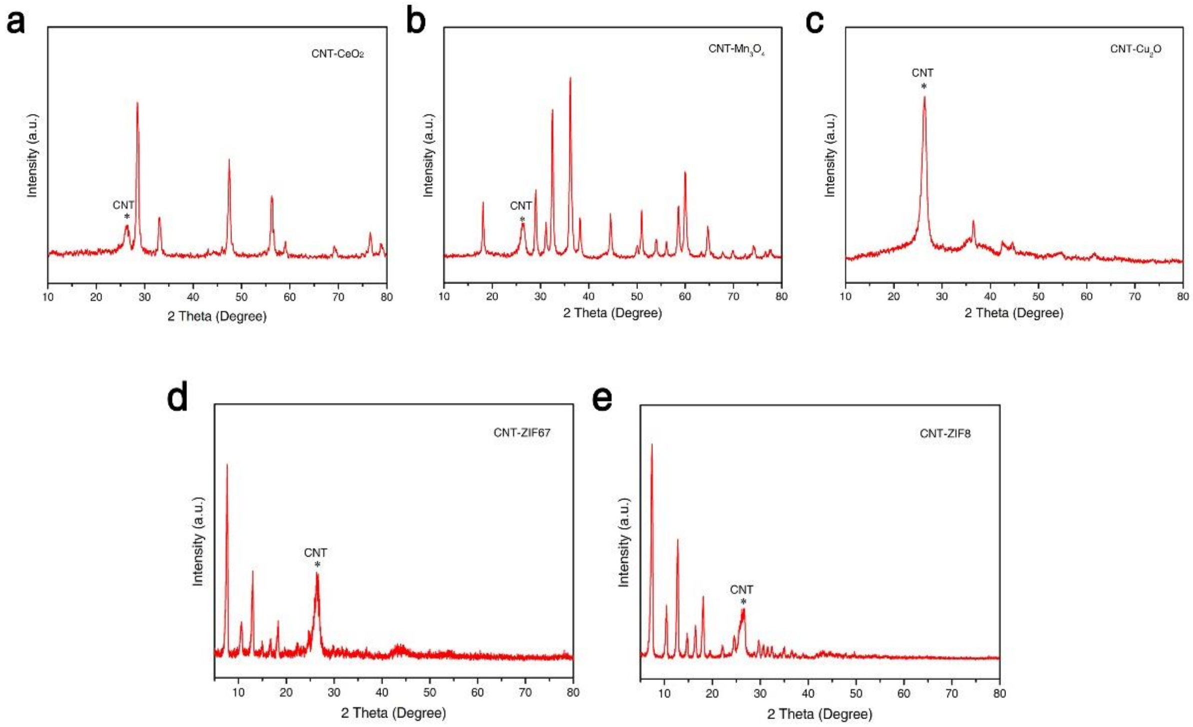

Supplementary Figure 2 The phase structure of other CNT/semiconductor composites. XRD patterns of CNT-CeO_2 (a), CNT-Mn_3O_4 (b), CNT-Cu_2O (c), CNT-ZIF-67 (d), and CNT-ZIF-8 (e).

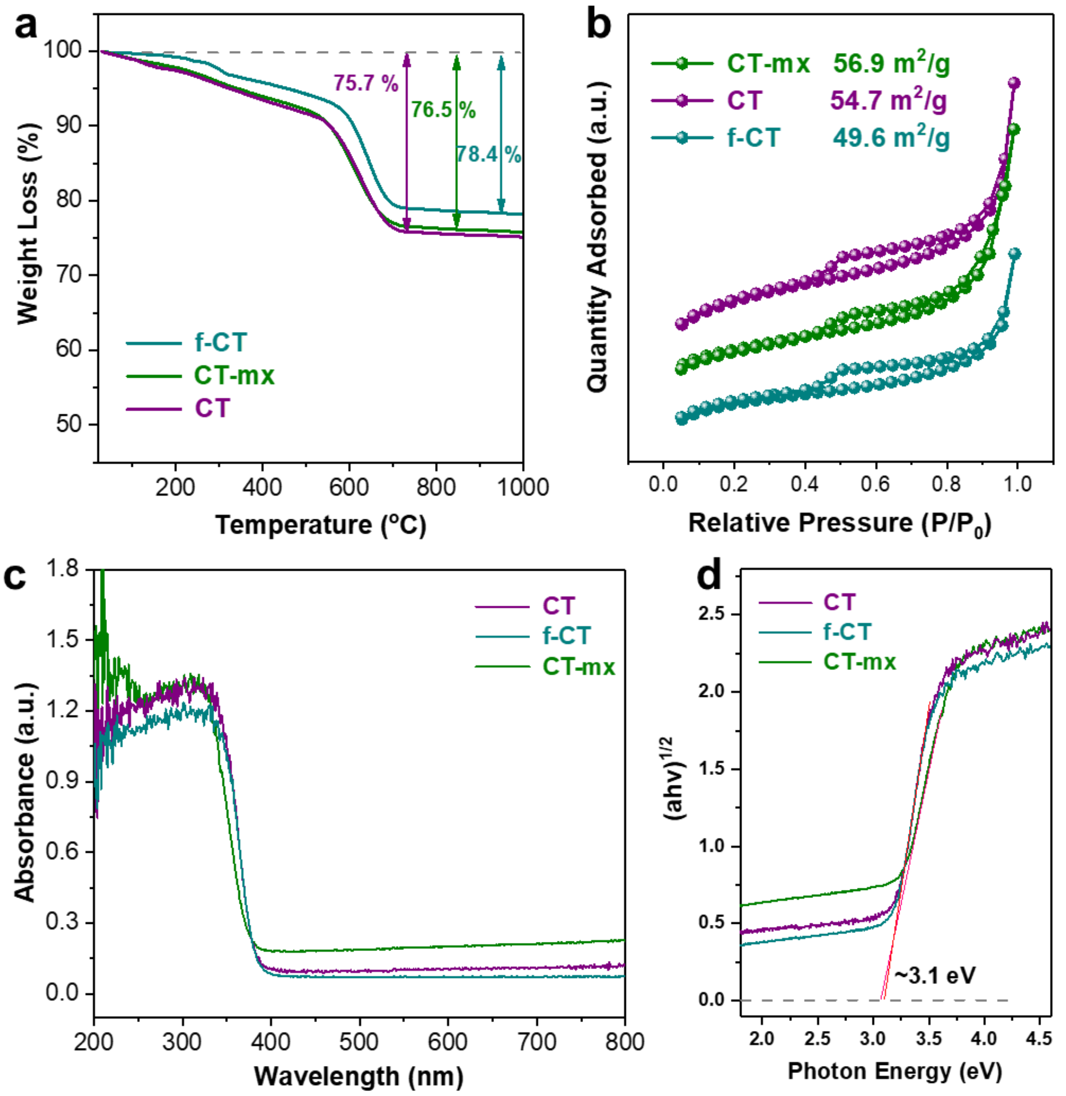

Supplementary Figure 3 The texture properties and optical properties over different samples. (a) Thermogravimetric analysis (TGA) curves in air, (b) nitrogen adsorption and desorption isotherm, (c) UV-vis spectra and (d) calculated band gap energies by Kubelka-Munk transformation of samples CT, f-CT and CT-mx.

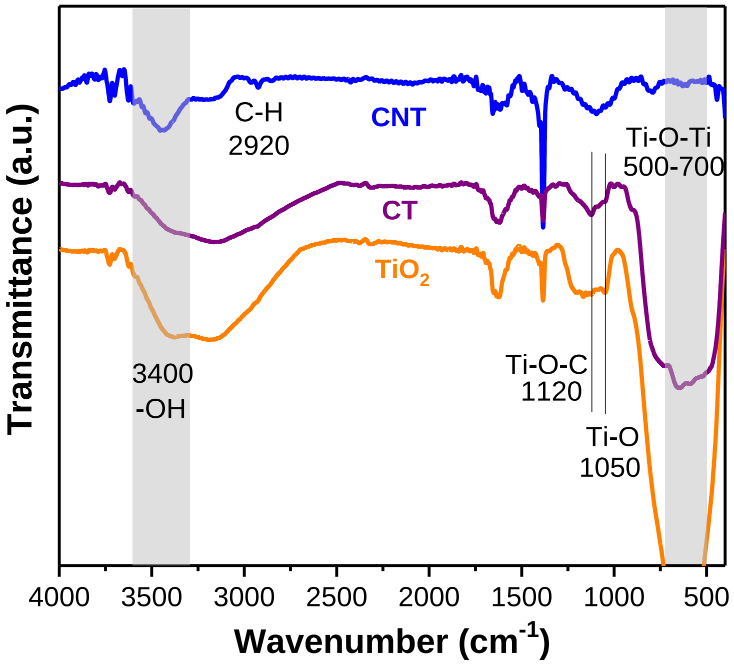

Supplementary Figure 4 The FT-IR spectra of sample CT, pure TiO_2 and CNT.

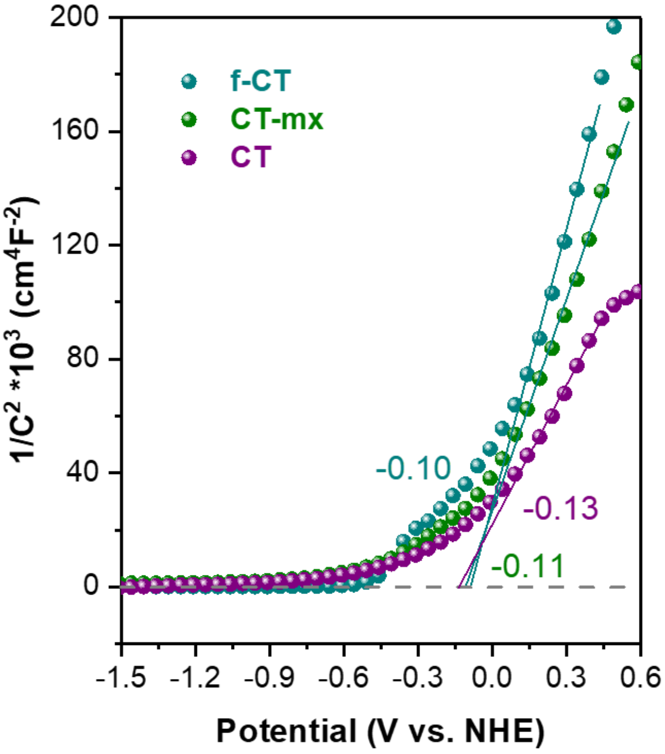

Supplementary Figure 5 Mott-Schottky plots of samples CT, f-CT and CT-mx.

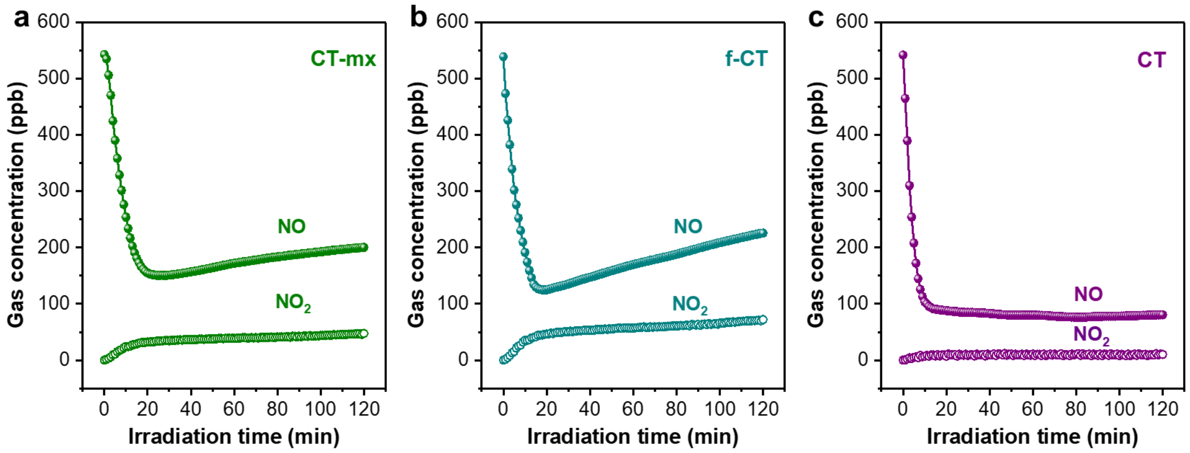

Supplementary Figure 6 The photocatalytic performance over different samples. The concentration profiles of NO and NO_2 during the photocatalytic NO oxidation reaction when different catalysts were applied (a) CT-mx, (b) f-CT and (c) CT.

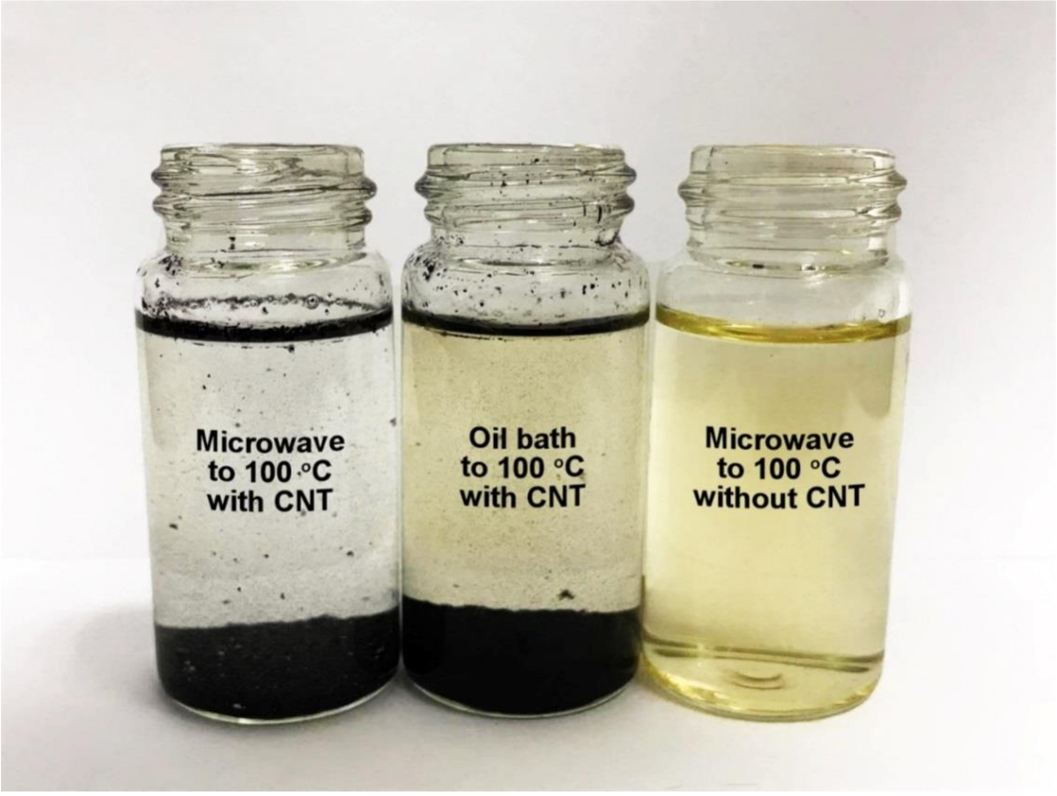

Supplementary Figure 7 The pictures show the important role of microwave irradiation in the presence of CNTs. Comparison of the reaction by microwave heating to 100°C for 10 min in DMSO solution containing $TiCl_3$ with (left), without CNTs (right) and by oil bath heating to 100 °C for 10 min in DMSO solution containing $TiCl_3$ with CNTs (middle).

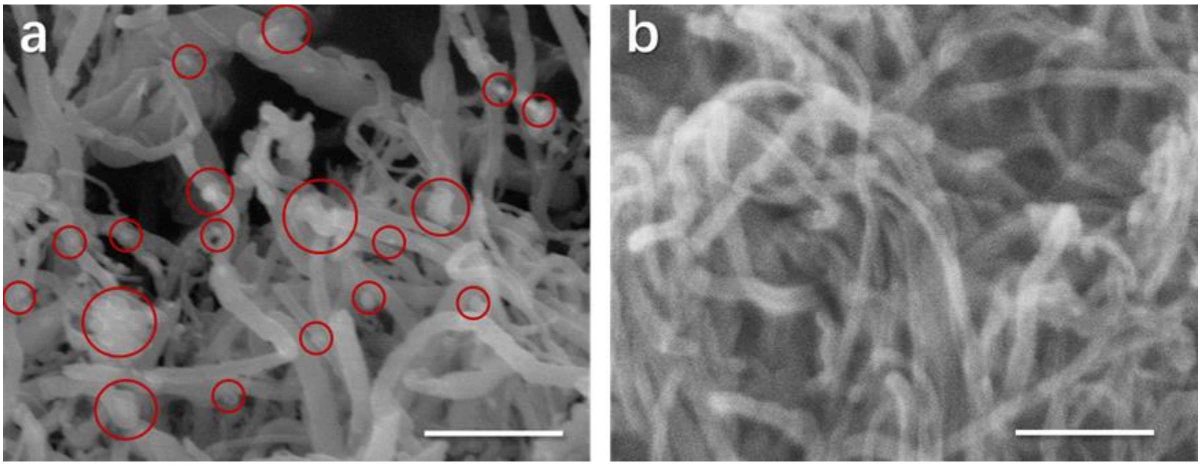

Supplementary Figure 8 $TiCl_3$ hydrolysis difference by microwave and oil bath. The FESEM images of CNT in $TiCl_3$ DMSO heated to 100 °C by microwave (a) and oil bath (b). Scale bars in (a) and (b): 500 nm.

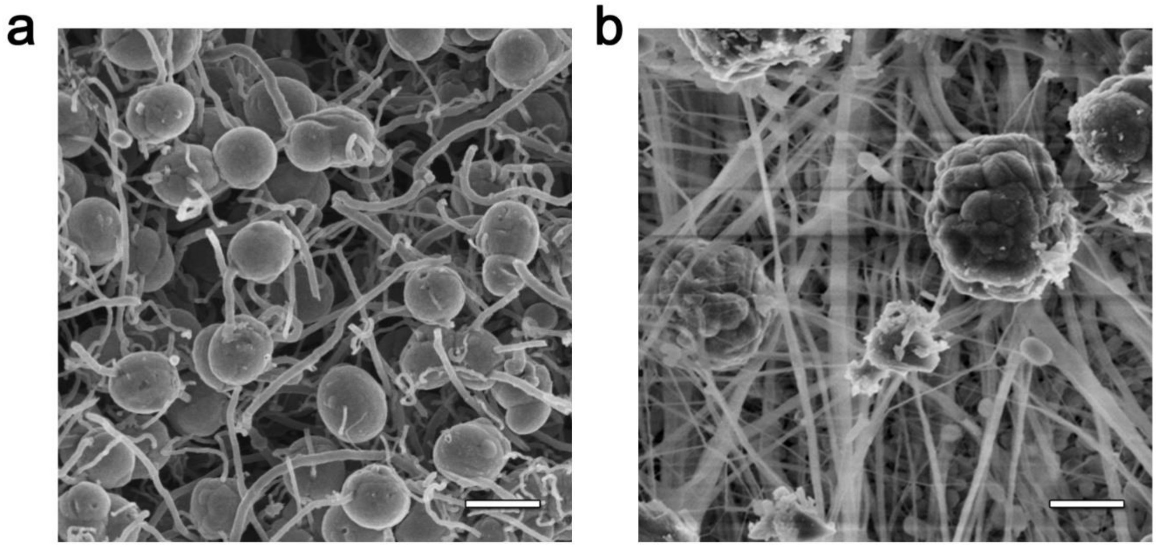

Supplementary Figure 9 Microwave induced super-hot dots on CNTs rather than PTFE. FESEM images of the TiO_2 obtained by microwave syntheses in the presence of (a) CNTs and (b) polytetrafluoroethylene (PTFE). Scale bars in (a) and (b): 1 μm.

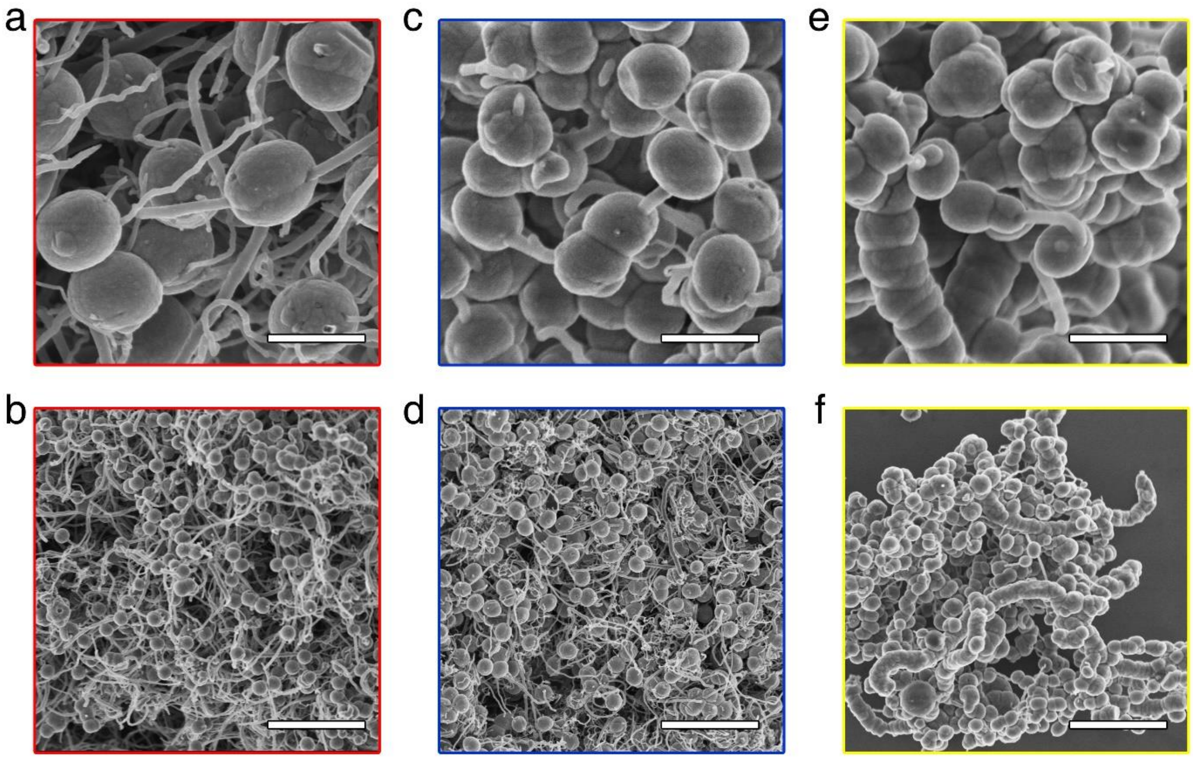

Supplementary Figure 10 Continuously tunable density of TiO_2 nanospheres threaded by CNTs. FESEM images of CNT-TiO_2 obtained by using CNTs pre-treated with HNO_3 for (a, b) 0 min, (c, d) 60 min, and (e, f) 240 min. Scale bars in (a) (c) (e): 1 μm, in (b) (d) (f) 5 μm.

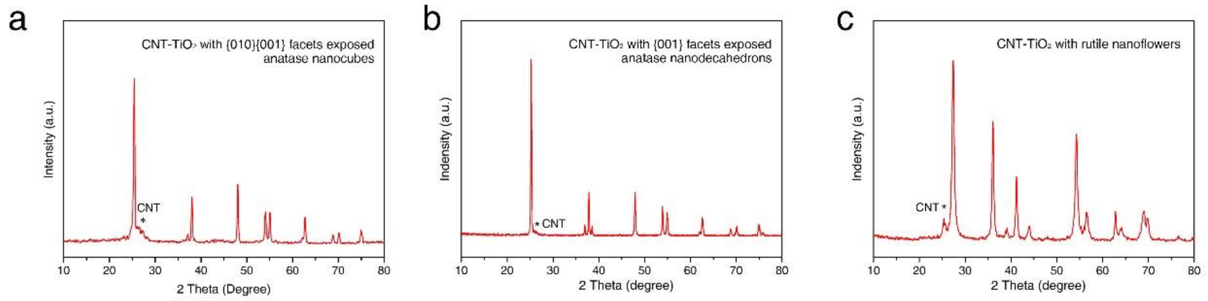

Supplementary Figure 11 XRD patterns of CNT-TiO_2 samples synthesized by using untreated CNTs. (a) CNTs-strung anatase TiO_2 nanocubes with (010) and (001) facets exposed, (b) CNTs-strung anatase TiO_2 nanodecahedrons with (001) and (101) facets exposed, and (c) CNTs-strung rutile TiO_2 nanoflowers.

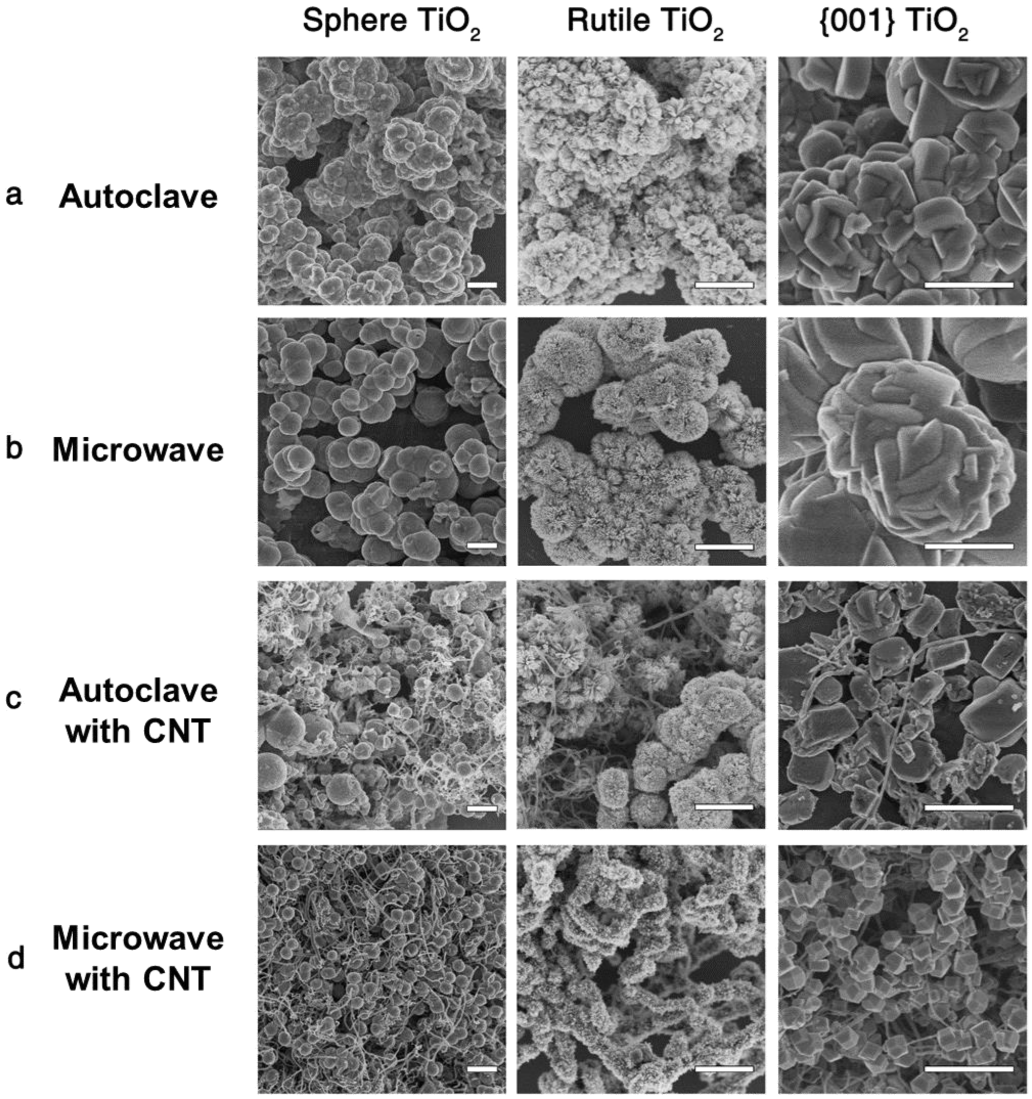

Supplementary Figure 12 Morphology differences by different reaction condition. FESEM images of anatase TiO_2 microspheres (left-column), rutile TiO_2 nanoflowers (middle-column), (001) and (101) facets exposed anatase TiO_2 nanodecahedrons (right-column) synthesized by autoclave hydrothermal heating without and with adding CNTs (a, c), microwave heating without and with adding CNTs (b, d). Scale bars 500 nm.

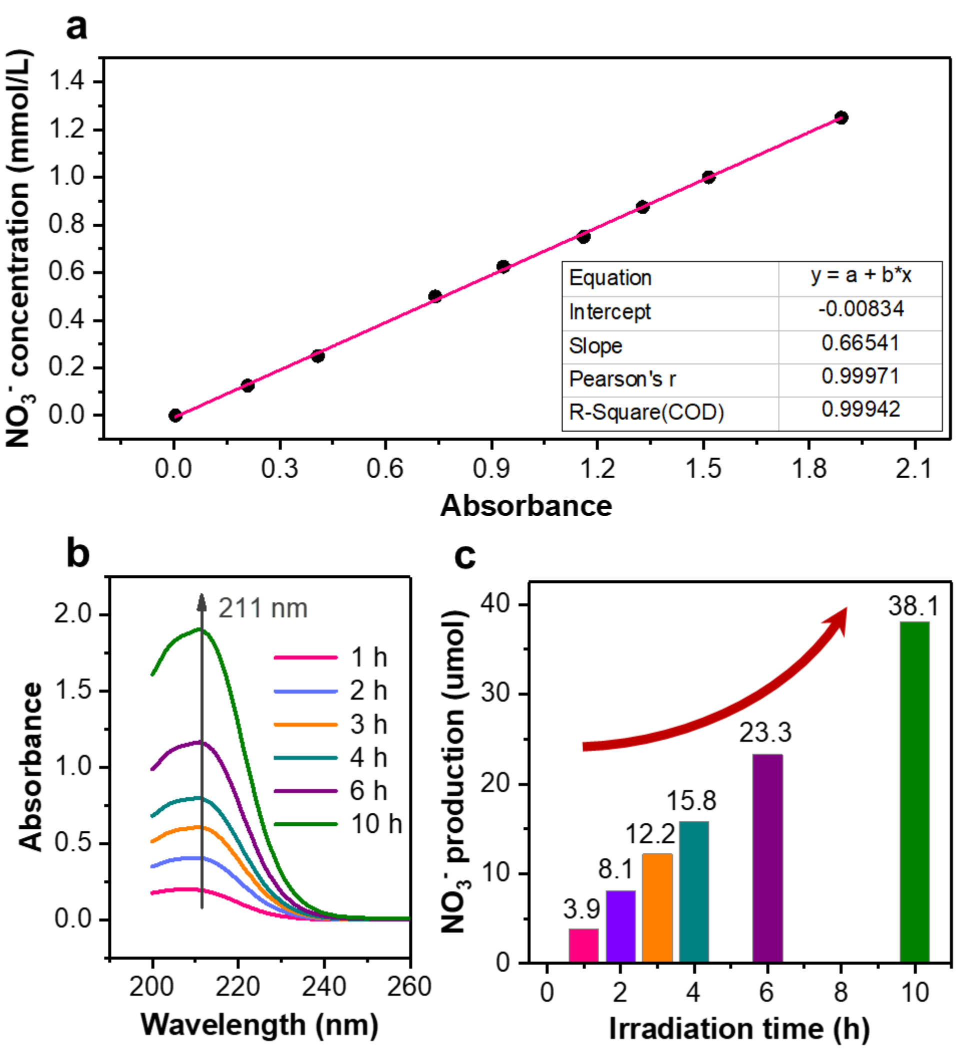

Supplementary Figure 13 Detection and calibration of reaction product NO_3^-. (a) Linear fitting curve of the NO_3^- concentration vs absorbance from liquid UV-vis spectra, (b) liquid UV-vis spectra of the catalysts washed solution with the increasing reaction time and (c) the NO_3^- production for the NO oxidation reaction.

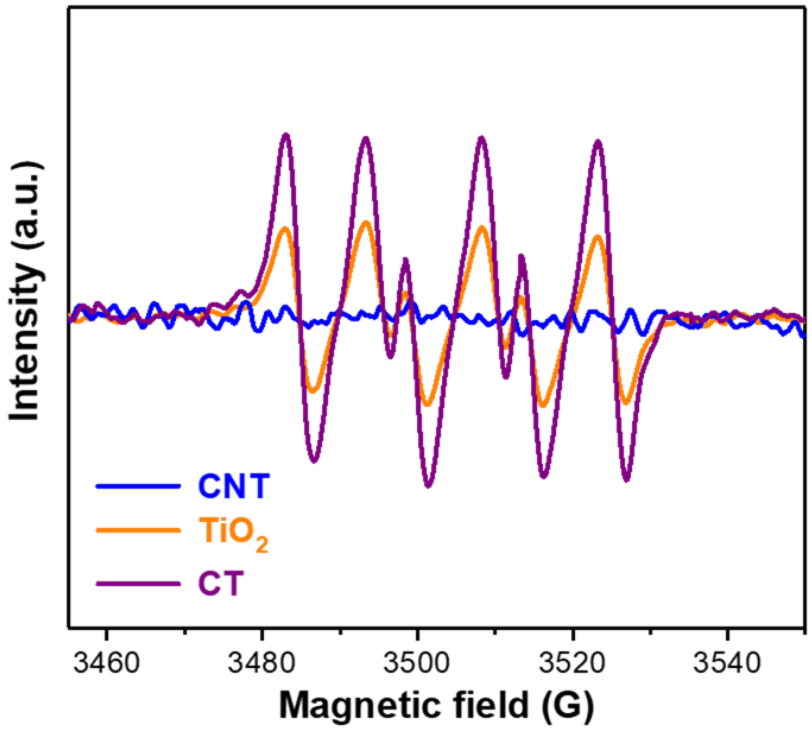

Supplementary Figure 14 DMPO spin-trapping ESR spectra in methanol dispersion for DMPO-•O_2^-.

Supplementary Table 1 The detail fitted parameters obtained from decay curves of samples.

Sample	A_1 (%)	τ_1 (ns)	A_2 (%)	τ_2 (ns)	τ_a (ns)
CT	87	2.4	13	16.0	9.2
CT-mx	84	3.7	16	22.3	13.6
f-CT	85	4.1	15	24.7	14.8

Supplementary Note 1

As shown in the TGA plot in Supplementary Figure 3a, the CNTs content in CT, CT-mx and f-CT was nearly the same, indicating the same TiO_2/CNTs ratio for fairly comparing their photocatalytic NO oxidation activity. Supplementary Figure 3b also displayed the similar nitrogen adsorption and desorption isotherm of the three samples. Furthermore, the three samples showed the similar light absorption since the absorption edge was located at ~380 nm corresponding to the similar band gap of ~ 3.1 eV from the Kubelka-Munk calculation (shown in Supplementary Figure 3c, d). Considering that these samples own the similar CNT/TiO_2 ratio, surface area and light absorption properties analyzed in Supplementary Figure 3, the difference in photocatalytic performance could be considered as their different charge transport properties.

Supplementary Note 2

The Fourier transform infrared (FT-IR) spectroscopy was introduced to find out the bond structure of the samples. As shown in Supplementary Figure 4, the fundamental vibrations of TiO_2 appeared in the 400-1100 cm^{-1} range. The intensive broad bands were ascribed to the stretching vibrations of Ti–O–Ti bonds (500-700 cm^{-1}) and Ti–O bonds (1050 cm^{-1}). When TiO_2 was threaded by CNT, some covalent bonds (Ti–O–C=O or Ti–O–C, located at ~1120 cm^{-1}) formed due to the esterification process between the CNTs surface carboxyl/hydroxyl groups and TiO_2.[1] As a result of the surface interaction between TiO_2 and CNT, the fundamental vibrations of TiO_2 (500–700 and 1050 cm^{-1}) were significantly weakened for CT sample relative to pure TiO_2. Such FTIR results confirmed the chemically bonded interfacial contact between CNTs and TiO_2 after the microwave hydrothermal reaction.

Supplementary Note 3

The charge dynamics of different samples was measured by the time resolved fluorescence spectra as presented in Fig. 3g. According to their charge generation, recombination and transfer processes, their decay curves could be fitted by the following exponential equation:

$$I_t = I_0 + A_1 \exp\left(-\frac{t}{\tau_1}\right) + A_2 \exp\left(-\frac{t}{\tau_2}\right) \qquad (1)$$

Where I_0 represents the baseline correction value, A_1 and A_2 are thepre-exponential factors, τ_1 and τ_2 are the lifetime (ns) of the process ofradiation and charge transfer, respectively. The average lifetime (τ_a) canbe calculated according to the following equation:

$$\tau_a = \frac{(A_1\tau_1^2 + A_2\tau_2^2)}{(A_1\tau_1 + A_2\tau_2)} \qquad (2)$$

The detailed fitted parameters were listed in Supplementary Table 1.

Supplementary Note 4

The plots shown in Supplementary Figure 5 were determined to obtain the flat-band potential and carrier density. As displayed in Supplementary Figure 5, the three samples have the similar flat band energy corresponding to the similar conduction band energy of -0.10~-0.13 eV. In addition, the capacitance measurement was performed on the electrode/electrolyte according to the Mott-Schottky equation:[2]

$$\frac{1}{C^2} = \frac{2}{N_D e \varepsilon_0 \varepsilon}\left(E - E_{FB} - \frac{kT}{e}\right) \qquad (3)$$

Where C is the space charge capacitance in the semiconductor, N_D is the electron carrier density, e is the elemental charge, ε_0 is the permittivity of a vacuum, ε is the relative permittivity of the semiconductor, E is the applied potential, E_{FB} is the flat band potential, T is the temperature, and *k* is the Boltzmann constant.

$$N_D = \frac{2}{e\varepsilon_0\varepsilon}\left(\frac{dE}{d\left(\frac{1}{C^2}\right)}\right) \quad (4)$$

From Supplementary Figure 5, with e = 1.6 × 10^{-19}C, ε_0= 8.86 × 10^{-12} F/m, ε= 48 for TiO_2 (anatase), we can calculate the photocarrier density which are 1.9*10^{25}, 1.2*10^{25} and 8.2*10^{24} cm^{-3} for CT, CT-mx and f-CT, respectively according to the above formula. In the equation, N_D reflects the photocarrier density; e refers to the elemental charge value; ε_0 is the vacuum permittivity while ε is the semiconductor permittivity; E is the applied potential and the C represents the space charge capacitance. This result proved that chloroplast structure significantly enhanced the carrier density and the charge separation of electron and holes, thus leading to the better catalytic performances.

Supplementary Note 5

The main photocatalytic oxidation product of NO is NO_3^- in this catalytic system which can be confirmed by detected NO_3^- on catalysts surface. The stability and selectivty of the catalysts mainly affected by the efficiency of photogenerated electron and holes according to the reaction equation listed as the following:

$$TiO_2 + hv \rightarrow e^- + h^+ \quad (5)$$

$$e^- + O_2 \rightarrow \cdot O_2^- \quad (6)$$

$$\cdot O_2^- + NO \rightarrow NO_3^- \quad (7)$$

$$NO + h^+ + O_2 \rightarrow NO_2 \quad (8)$$

$$NO_2 + h^+ + O_2 \rightarrow NO_3^- \quad (9)$$

Here, the amount of the holes determined the oxidation selectivity of NO. Fast carrier separation kinetic promoted the high concentration of reactive holes to realize deeply oxidation to NO_3^- instead of NO_2. By checking the real-time NO and NO_2 concentration in Fig. S6, the NO_2 selectivity in 2 h could be 4.5 %, 6.8% and 1.2% for CT-mx, f-CT and CT,

respectively. They are coincident with the charge separation efficiency according to the EIS, photoluminescence decay and carrier's density results.

Supplementary Note 6

The main product in this photocatalytic oxidation system can be confirmed by the detected NO_3^- species on the catalysts surface. The detailed test process was described as the following. 0.2 g of the CT catalysts after different reaction time were collected and ultrasonicated in 20 mL of ultrapure water to completely dissolve the surface adsorbed HNO_3. After centrifugation, 1 mL of the washed solution was added to 1 mL of HCl (aq., 1M) and 1 mL of ammonium sulfamate (aq., 50 g L^{-1}). Then, it was diluted to 20 mL in a volumetric flask. The absorbance intensity was analyzed by a UV-vis spectrophotometer (UV7504/PC). After substituted the absorbance intensities located at 211 nm (Supplementary Figure 13b) into the fitted standard curve shown in Supplementary Figure 13a, the NO_3^- production could be obtained. Displayed in Supplementary Figure 13c, the NO_3^- production increased with the irradiation time. After calculation, the NO_3^- selectivity could be more than 98 % over the sample CT. The stability of the catalyst mainly affected by the efficiency of photogenerated electron and holes according to the reaction equation as mentioned above. Herein, the amount of the holes determined the NO oxidation selectivity. Fast carrier separation kinetics promoted the high concentration of reactive holes to realize deeply oxidation of NO to NO_3^- instead of NO_2.

Supplementary Note 7

To get fully understand of the reaction mechanism, we recorded electron spin resonance (ESR) signals of radical's spin-trapped by 5, 5'-dimethyl-1-pyrroline-N-oxide (DMPO) in methanol to detect DMPO-$\bullet O_2^-$ shown in Supplementary Figure 14. Under the UV irradiation, the CNT showed no ESR signal, since CNT is not a semiconductor and could not produce $\bullet O_2^-$ via photocatalytic route. On the contrary, TiO_2 showed signals of DMPO-$\bullet O_2^-$ indicating

the formation of $\bullet O_2^-$ generated by conduction band electron. The CT sample displayed an enhanced intensity on the DMPO-$\bullet O_2^-$ signals due to the fast kinetic of electron transport from TiO_2 to the CNT in this chloroplast structure.

Supplementary Methods

Synthesis of CNT-TiO_2 with (010) and (001) facets exposed anatase nanocubes

The CNTs (with diameter in 60–100 nm, length in 5–15 μm) used in the synthesis were bought from the Shenzhen Nanoport company. In a typical synthesis, 20 mg CNTs were dispersed in 18 mL ultrapure water for 30 min to form a black suspension. Subsequently, 2 mL 15 wt% $TiCl_3$ aqueous solution with 10~15 wt% HCl was added to the suspension with magnetic stirring for 10 min, followed by adding 0.10 mL of ionic liquid 1-butyl-3-methylimidazolium tetrafluoroborate (Bmim[BF_4], Shanghai Cheng Jie Chemical Co. Ltd) and 0.2 g of sodium dodecyl benzene sulfonate (SBDS) within 10 min under magnetic stirring. Then, all the mixture was moved to a 40 ml quartz vessel with a Teflon lid. It was treated at 120 °C for 30 min with a heating rate of 15 °C min^{-1} and with an initial pressure of 35 bar by N_2 in a single chamber microwave digestion system. Finally, the resulting powder was washed with deionized water and absolute ethanol for 3 times, followed by vacuum drying at 80 °C for 4 h.

Synthesis of CNT-TiO_2 with (001) facets and (101) facets exposed anatase nanodecahedrons

In a typical synthesis, 20 mg CNTs were dispersed in 17 mL ultrapure water for 30 min to form a black suspension, followed by adding 2 mL 15 wt% $TiCl_3$ aqueous solution with 10~15 wt% HCl within 10 min under magnetic stirring. Subsequently, 1.0 mL of ionic liquid 1-methyl-imidazolium tetrafluoroborate (Hmim[BF_4], Shanghai Cheng Jie Chemical Co. Ltd) was added within 10 min under magnetic stirring. All the mixture was moved to a 40 ml quartz vessel with a Teflon lid. It was treated at 150 °C for 30 min with a heating rate of 15 °C min^{-1} and with an initial pressure of 35 bar by N_2 in a single chamber microwave digestion system. After the reaction, the resulting powder was washed with deionized water and absolute ethanol for 3 times and dried in a vacuum at 80 °C for 4 h.

Synthesis of CNT-TiO_2 with rutile nanoflowers

In a typical synthesis, 20 mg CNTs were dispersed in 37 mL ultrapure water for 30 min to form a black suspension. And then, 3.0 mL 15 wt% $TiCl_3$ aqueous solution with 10~15 wt% HCl was added within 10 min under magnetic stirring. All the mixture was moved to a 50 ml Teflon vessel. It was treated at 150 °C for 30 min with a heating rate of 15 °C min^{-1} by microwave heating system. After the reaction, the resulting powder was washed with deionized water and absolute ethanol for 3 times and dried in a vacuum at 80 °C for 4 h.

Synthesis of CNT-Cu_2O nanocomposite

In a typical synthesis, 30 mg $Cu(OAc)_2$ (Aladdin) and 10 mg CNTs were dispersed in 36 mL ethylene glycol (Sinopharm Group Co. Ltd), and then add 4 mL water to the system with magnetic stirring for 10 min. All the mixture was moved to a 40 mL quartz vessel with a Teflon lid. It was treated at 150 °C for 10 min with a heating rate of 15 °C min^{-1} and with an initial pressure of 35 bar by N_2 in a single chamber microwave digestion system (Ultrawave, Milestone). After the reaction, the resulting powder was washed with deionized water and absolute ethanol for 3 times and dried in a vacuum at 80 °C for 4 h.

Synthesis of CNT-Mn_3O_4 nanocomposite

In a typical synthesis, 20 mg CNTs were dispersed in 25 mL ethylene glycol (Sinopharm Group Co. Ltd) containing 0.20 g Polyvinylpyrrolidone (K-30, Aladdin) for 10 min by ultra-sonic cleaner. And then, 1.0 mol $Mn(OAc)_2$ (Aladdin) were added to the reactant system with magnetic stirring for 10 min. All the mixture was moved to a 40 mL quartz vessel with a Teflon lid. It was treated at 195 °C for 10 min with a heating rate of 15 °C min^{-1} and with an initial pressure of 35 bar by N_2 in a single chamber microwave digestion system (Ultrawave, Milestone). After the reaction, the resulting powder was washed with deionized water and absolute ethanol for 3 times and dried in a vacuum at 80 °C for 4 h.

Synthesis of CNT-CeO_2 nanocomposite

In a typical synthesis, 10 mg CNTs were dispersed in 8 mL ultrapure water for 10 mins by ultra-sonic cleaner. And then, 2 mL $Ce(NO_3)_3$ (Aladdin) (0.2 M) solution was added with magnetic stirring for 10 mins. Next, 1 mL ionic liquid 1-methyl-imidazolium tetrafluoroborate (Hmim[BF_4] , Shanghai Cheng Jie Chemical Co. Ltd) was added into the reactant system with magnetic stirring for another 10 mins. All the mixture was moved to a 40 mL quartz vessel with a Teflon lid. It was treated at 150 °C for 30 min with a heating rate of 15 °C min^{-1} and with an initial pressure of 35 bar by N_2 in a single chamber microwave digestion system (Ultrawave, Milestone). After the reaction, the resulting powder was washed with deionized water and absolute ethanol for 3 times and dried in a vacuum at 80°C for 4 h.

Synthesis of CNT-ZIF-8 nanocomposite

In a typical synthesis, 100 mg carbon nanotubes without acid treatment were dispersed in 10 mL ultrapure water for 30 min to form a black suspension. And then, 0.11 g $Zn(OAc)_2·2H_2O$ (Sinopharm Group Co. Ltd) was added to the suspension with magnetic stirring for 10 min. After that, 0.41 g 2-Methylimidazole (Sigma-Aldrich) was added into the above solution. All the mixture was moved to a 40 mL quartz vessel with a Teflon lid. It was treated at 120 °C for 30 min with a heating rate of 15 °C min^{-1} and with an initial pressure of 35 bar by N_2 in a single chamber microwave digestion system (Ultrawave, Milestone). After the reaction, the resulting powder was washed with deionized water and absolute ethanol for 3 times and dried in a vacuum at 80 °C for 4 h.

.

Synthesis of CNT-ZIF-67 nanocomposite

In a typical synthesis, 100 mg carbon nanotubes without acid treatment were dispersed in 25 mL methanol (Sinopharm Group Co. Ltd) containing 0.33 g 2-Methylimidazole

(Sigma-Aldrich) for 30 min to form a black suspension. And then, 0.25 g $Co(NO_3)_2$ (Aladdin) was added to the suspension with magnetic stirring for 10 min. All the mixture was moved to a 40 mL quartz vessel with a Teflon lid. It was treated at 140 °C for 30 min with a heating rate of 15 °C min^{-1} and with an initial pressure of 35 bar by N_2 in a single chamber microwave digestion system (Ultrawave, Milestone). After the reaction, the resulting powder was washed with absolute ethanol for 3 times and dried in a vacuum at 80 °C for 4 h.

Acid treatment of CNTs

In the typical treatment route, 500 mg of the CNTs were suspended in 40 ml of concentrated nitric acid (HNO_3), keeping the temperature of 90 °C by oil bath and refluxed for 30, 60, 120 and 240 min. After washing with deionized water until the supernatant attained a pH around 7, the samples were dried under vacuum at 80 °C. The as-treated CNTs were referred thereof as CNT-A30, CNT-A60, CNT-A120and CNT-A240 depending on the acid treatment time (30, 60, 120 and 240 min, respectively).

Microwave Synthesis of reference samples without CNTs

Samples without CNTs as references were synthesized by microwave-assisted method involving the same reaction mixture without CNTs.

Synthesis of reference samples f-CT

f-CT was synthesized by the similar route of CT by using CNT-A240 instead of the untreated CNT. In a typical synthesis, 20 mg CNT-A240 were dispersed in 18 mL dimethyl sulfoxide (DMSO, Aladdin) for 30 min to form a black suspension in an ultrasonic cleaner. Then, 2 mL 15 wt% $TiCl_3$ aqueous solution with 10~15 wt% HCl (Merck-Schuchardt) was added to the suspension with magnetic stirring for 10 min. Subsequently, the mixture was moved to a 40 ml quartz vessel with a Teflon lid. It was treated at 180 °C for 30 min with a heating rate of

15 °C min^{-1} and with an initial pressure of 35 bar by N_2 in a single chamber microwave digestion system (Ultrawave, Milestone). The resulting powder was washed with deionized water and absolute ethanol for 3 times, followed by vacuum drying at 80 °C for 4 h.

Synthesis of reference samples CT-mx

CT-mx was prepared by mechanical mixing the CNT and pure TiO_2 according to the CNT/TiO_2 mass ratio in sample CT.

Hydrothermal Synthesis of reference samples

As references, all the samples obtained by the microwave-assisted method were also synthesized by the hydrothermal method maintaining the same temperature but different reaction time. If it takes 30 min to synthesize samples by the microwave-assisted method, it would heat for 6 h by the hydrothermal method. Also, samples without CNTs as references were synthesized by the hydrothermal method using the same reaction condition just without adding CNTs.

Temperature tracking measurement

The solution temperature tracking measurement was to test the temperature raising rate of solution under different conditions. Here, 50 mL of DMSO was used as the solution, 50 mg of CNT with different acid-treatment time and 50 mg of PTFE fibers were added to the solution. After well dispersion by ultrasonication, the solution was transferred to a quartz reaction tube. Bare DMSO solution was also put into a quartz tube as the reference sample. For the microwave heating test, the quartz tube was placed in the center of the microwave workstation (Sineo MDS II), and 800 W microwave with 2.45 GHz frequency was applied. An infrared detector located ~10 cm above the solution was used to detect the real-time temperature of the solution. For the oil-bath test, the quartz tube was inserted into 90 °C of methyl silicone with

the hot plate to keep this temperature. A thermocouple thermometer (IKA ETS-D6) was inserted into the DMSO solution to detect the real-time temperature. Every 10 s, we read and record the temperature data from the thermocouple or the infrared detector.

Characterization

The crystal phase was determined by X-ray diffraction (XRD, Rigaku Dmax-3C Cu-Ka). Particle size and morphologies were observed on a transmission electron microscopy (TEM, JEOL-2010F, 200 kV) and a field scanning electron microscopy (FESEM, HITACHI, S-4800). Photoelectrochemical measurements were carried out in a conventional three-electrode, single-compartment quartz cell on an electrochemical station (CHI 660D). The samples with an active area of *ca.*4.0 cm^2 on FTO glass were served as working electrode. The counter electrode and the reference electrode were platinum sheet and saturated calomel electrode (SCE), respectively. A bias voltage of 0.50 V was utilized for driven the photo-generated electrons transfer from working electrode to platinum electrode. A 3 W LED light located at 10 cm away from the photoelectrochemical cell was used as light sources (wavelength = 365 nm). A 0.20 M Na_2SO_4 aqueous solution was used as the electrolyte. The EIS tests were carried out at the bias of the open circuit voltage and recorded over a frequency ranged from 0.1 to 10^5 Hz with AC amplitude of 10 mV. Thermal gravimetric analysis (TGA) was performed by the PerkinElmer Pyris Diamond TG analyzer under air with a 5 °C min^{-1} heating ramp. The ns-PL measurements were performed on a LP-920 Laser flash photolysis setup (Edinburgh Instruments). The 355 nm pump laser pulse was obtained from the third harmonic output of an Nd:YAG Q-switched laser. The signals were analyzed by a symmetrical Czerny−Turner monochromator, detected by a Hamamatsu R928 photomultiplier, and processed via an interfaced computer and analytical software. This ns-PL experiment was carried out in air-saturated aqueous solutions. At the sample position, the average powers were about 70~80 mW for the pump beam. The UV-Vis diffuse reflectance spectra (DRS)

were obtained on a UV-Vis spectrophotometer (UV-Vis DRS, Shimadzu UV-2450). The specific surface areas (S_{BET}) of samples were tested by nitrogen adsorption and desorption isotherm and calculated by applying Brunauer–Emmett–Teller (BET) models on desorption branches (Micromeritics, TriStar II 3020). The electron spin resonance (ESR) spectra were obtained at 100 K using a Bruker EMX-8/2.7 ESR spectrometer. Fourier transform infrared (FT-IR) spectra were obtained on a Nicolet Magna 550 spectrometer using the KBr method.

Supplementary references

1. Yu J., Ma T. & Liu S. Enhanced photocatalytic activity of mesoporous TiO_2 aggregates by embedding carbon nanotubes as electron-transfer channel. *Phy. Chem. Chem. Phy.* **8**, 3491–3501 (2011).
2. Ye M. , Gong J., Lai Y., Lin C. & Lin Z. High-efficiency photoelectrocatalytic hydrogen generation enabled by palladium quantum dots-sensitized TiO_2 nanotube arrays. *J. Am. Chem. Soc.* **134**, 15720–15723 (2012).
